# A Cross-Sectional Study of the Impact of the COVID-19 Pandemic and Lockdown Orders on Injury Prevalence in National Collegiate Athletic Association (NCAA) Division I Athletes

**DOI:** 10.7759/cureus.38296

**Published:** 2023-04-29

**Authors:** Carly Heffernan, Maddison McLellan, Jason Xu, John Billimek, Brian Y Kim

**Affiliations:** 1 Family Medicine, University of California (UC) Irvine School of Medicine, Irvine, USA; 2 Orthopedic Surgery, University of California, Irvine, USA; 3 Family Medicine, University of California (UC) San Diego Health, San Diego, USA

**Keywords:** covid-19 pandemic, injury, ncaa athlete, sports medicine, covid-19

## Abstract

Objective

The purpose of this study was to analyze the effect of the coronavirus disease 2019 (COVID-19) on injury prevalence in National Collegiate Athletic Association (NCAA) varsity athletes following mandatory state-issued stay-at-home orders in March 2020. A secondary objective was to evaluate the relationship between COVID-19 infection and injury prevalence.

Methods

The respondents were recruited during pre-participation evaluations held at a Division I university in California in the summer of 2021, as well as via emails shared by athletic trainers at the institution. Data was collected using the Qualtrics Survey Platform (Qualtrics, Provo, UT). For all questions regarding the effects of the COVID-19 pandemic, the participants were asked to compare March 2020-March 2021 ("post-pandemic") to March 2019-March 2020 ("pre-pandemic"). Injury was defined as a physical complaint or condition sustained by an athlete during participation in training or competition that resulted in at least one missed day of practice or competition. The study participants were also asked to disclose their history of laboratory-confirmed COVID-19 infection.

Results

One hundred forty-six respondents completed the survey, with a 72.3% response rate. Of the respondents, 33.6% (n=49) reported sustaining at least one injury in the year preceding the pandemic, whereas 45.2% (n=66) of respondents reported sustaining at least one injury within the first year of the pandemic, a 34.5% relative increase in injuries (RR=1.35; 95% CI=1.01, 1.80). There was no significant difference in the number of upper body (RR=1.64; 95% CI=0.8, 3.34; p=0.177) versus lower body (RR=1.31; 95% CI=0.94, 1.82; p=0.11) injuries before and after the pandemic onset. Thirty-two respondents reported a history of COVID-19 infection. The athletes who reported a prior COVID-19 diagnosis were no more likely than the athletes with no prior COVID-19 diagnosis to obtain an injury from March 2020 to February 2021 (p=0.85).

Conclusion

This study indicates that the COVID-19 pandemic and stay-at-home orders were associated with a greater risk of injury in this cohort of collegiate athletes. Interestingly, a history of laboratory-confirmed COVID-19 infection was not associated with increased risk of injury.

## Introduction

On March 4, 2020, the governor of California proclaimed a state of emergency in response to the threat of coronavirus disease 2019 (COVID-19). In the days that followed, the National Collegiate Athletic Association (NCAA) cancelled all collegiate sports championships for the remainder of the academic year [1], and the state of California issued mandatory stay-at-home orders. These decisions, and the effects of the pandemic that followed, were a significant disruption to the landscape of college athletics and limited practice frequency, access to sports equipment, and access to injury prevention and rehabilitation resources.

Previous studies examining injury prevalence during gaps in official practice have shown conflicting results. Orhant et al. found no changes in injury occurrence or patterns in French professional soccer clubs during the disrupted 2020/2021 season compared to the season prior [2]. In separate studies, Platt et al. [3] and Platt et al. [4] noted no significant changes in injury incidence in the National Football League (NFL) in 2020 compared to the 2018-2019 season, though injury rates during the 2020 Major League Baseball (MLB) season were almost twice that of the two seasons prior. Notably, the subjects in the studies of Orhant et al. [2], Platt et al. [3], and Platt et al. [4] continued to train on their own during the lockdown periods, which may downplay the complete role of deconditioning on injury incidence following the stay-at-home orders. In the general population, a study by Keays et al. found a decrease in sports-related pediatric injuries following the onset of the COVID-19 pandemic [5].

The availability of resources to perform at-home training during disruptions in sports is variable, with a survey of 401 collegiate athletes reporting that only 37.8% of student-athletes could fully perform their training activities following the COVID-19 pandemic [6]. This lack of access could be particularly detrimental to athletes engaging in rehabilitation programs after injury or surgery [7,8]. Return to sport after a disruption in routine training also has its risks, with data from the NFL demonstrating an increase in anterior cruciate ligament (ACL) tears following a player lockout [9].

The purpose of this study was to analyze the effect of the COVID-19 pandemic and mandatory stay-at-home orders on injury prevalence and characteristics in varsity Division I athletes. We hypothesized that practice and competition disruptions due to COVID-19 would lead to increased injury rates compared to the previous year and that a history of COVID-19 infection would confer an increased risk of injury. This article was previously presented as a meeting abstract at the American Medical Society for Sports Medicine (AMSSM) on April 11, 2022.

## Materials and methods

Study subjects were recruited during three collegiate pre-participation physicals held in the summer of 2021 and through emails distributed by team athletic trainers. Surveys were accessed through quick response (QR) codes posted at pre-participation physical locations and included in emails. The response rate was calculated as the number of completed surveys divided by the total number of times the survey was accessed and started. QR codes were not individualized, so we were unable to ascertain how many individuals received or accessed the survey. The inclusion criteria were (1) age of 18 years or older, (2) at least one year of varsity college sports experience, and (3) active status on the roster of an NCAA collegiate sport at the time of survey completion. Only returning collegiate athletes with at least one year of collegiate sports experience were included in our analysis. The participants from all varsity-level sports teams were included. Partially completed surveys were excluded from the final analysis. Study involvement was optional, and all participants gave informed consent before starting the anonymous survey. Our study was determined to be institutional review board (IRB)-exempt as participation involved the completion of virtual anonymous surveys.

Data was collected using a Qualtrics Survey Program (Qualtrics, Provo, UT). There was no control group used. All participants were asked to compare March 2020-March 2021 ("post-pandemic") to March 2019-March 2020 ("pre-pandemic") for all inquiries regarding the COVID-19 pandemic impact. The respondents were asked questions regarding the number of injuries, injury location, injury setting, and the type of injury during the pre-pandemic and post-pandemic time periods. An injury was defined as a physical complaint or condition sustained by an athlete during participation in a sports activity that resulted in at least one missed day of practice or competition. The participants were also asked to disclose any prior laboratory-confirmed COVID-19 diagnosis. Demographic data regarding age, year in collegiate sports, sports team, gender, and year in school was obtained for each participant. Group comparisons were performed using Fisher's exact test. Injury rate analysis involved comparing the number of respondents with at least one injury before and after the start of the COVID-19 pandemic.

## Results

The survey was accessed 202 times, and 146 surveys were completed, a response rate of 72.3%. Demographic data is provided in Table 1. A total of 77 females (52.4%) and 69 males (47.3%) completed the survey. The results comprised 55 sophomore respondents (37.7%), 39 junior respondents (26.7%), and 52 senior, fifth-year, or graduate respondents (35.6%). Overall, 16 sports teams were included in our study with the greatest number of respondents coming from the females' track and field (20 responses, 13.7%) and females' cross-country teams (19 responses, 13.0%). Females' volleyball made up the smallest portion of our participants (two responses, 1.4%). Thirty-two participants reported a history of laboratory-confirmed COVID-19 infection (21.9%).

**Table 1:** Demographic Information Participant demographics including gender, year in sport, history of confirmed COVID-19 infection, and sports team COVID-19: coronavirus disease 2019

Demographics	N (%)
	N=146 (100%)
Gender	
Female	77 (52.4%)
Male	69 (47.3)
Year	
Sophomore	55 (37.7%)
Junior	39 (26.7%)
Senior, Fifth-Year, or Graduate Student	52 (35.6%)
History of COVID-19 Infection	
Yes	32 (21.9%)
No	114 (78.1%)
Sport	
Males' Baseball	16 (11.0%)
Males' Basketball	6 (4.1%)
Males' Cross-Country	7 (4.8%)
Males' Golf	4 (2.7%)
Males' Soccer	4 (2.7%)
Males' Tennis	5 (3.4%)
Males' Track and Field	10 (6.8%)
Males' Volleyball	5 (3.4%)
Males' Water Polo	12 (8.2%)
Females' Cross-Country	19 (13.0%)
Females' Golf	4 (2.7%)
Females' Soccer	3 (2.1%)
Females' Tennis	8 (5.5%)
Females' Track and Field	20 (13.7%)
Females' Volleyball	2 (1.4%)
Females' Water Polo	13 (8.9%)

Of the respondents, 33.6% (n=49) reported sustaining at least one injury in the year preceding the pandemic, whereas 45.2% (n=66) of respondents reported sustaining at least one injury within the first year of the pandemic (RR=1.35; 95% CI=1.01, 1.80). Injury data is provided in Table 2. Due to a small number of reported injuries by location, injuries were further grouped into upper body (the shoulder, elbow, hand, and wrist) and lower body (the hip, knee, ankle, and foot) injuries. There was no statistically significant increase in the number of upper body or lower body injuries from before the COVID-19 lockdown started to after the lockdown measures began (upper body: RR=1.64; 95% CI=0.8, 3.34; p=0.177; lower body: RR=1.31; 95% CI=0.94, 1.82; p=0.11). Most injuries were sustained during official team practice both before (n=26, 17.8%) and after the start of the pandemic (n=37, 25.3%), compared to that during official competition (before pandemic: n=15, 10.3%; after the pandemic started: n=19, 13.0%), individual workouts provided by team coaches and staff (before pandemic: n=6, 4.1%; after the pandemic started: n=19, 13.0%), personal workouts (before pandemic: n=7, 4.8%; after the pandemic started: n=15, 10.3%), workouts with non-team coaches or staff (before pandemic: n=4; 2.7%; after the pandemic started: n=5, 3.4%), outside of sports (before pandemic: n=5, 3.4%; after the pandemic started: n=9, 6.2%), no injuries (before pandemic: n=21, 14.4%; after the pandemic started: n=13, 8.9%), or others (before pandemic: n=3, 2.1%; after the pandemic started: n=5, 3.4%).

**Table 2:** Injuries Injury prevalence increased from 33.6% (n=49) before the pandemic (March 2019-February 2020) to 45.2% (n=66) after the initiation of lockdown measures (March 2020-February 2021). There was no statistically significant increase in the number of upper body or lower body injuries from before the COVID-19 lockdown started to after the lockdown measures began (upper body: RR=1.64; 95% CI=0.8, 3.34; p=0.177; lower body: RR=1.31; 95% CI=0.94, 1.82; p=0.11) COVID-19: coronavirus disease 2019

	N (%)	RR	P
At Least One Injury			
Before COVID-19 Lockdowns	49 (33.6%)	1.35 (1.01, 1.80)	0.04
After COVID-19 Lockdowns	66 (45.2%)		
Upper Body Injuries			
Before COVID-19 Lockdowns	11 (17.5%)	1.64 (0.8, 3.34)	0.18
After COVID-19 Lockdowns	18 (20.5%)		
Lower Body Injuries			
Before COVID-19 Lockdowns	42 (66.7%)	1.31 (0.94, 1.82)	0.11
After COVID-19 Lockdowns	55 (62.5%)		

Athletes reporting a prior laboratory-confirmed COVID-19 diagnosis were no more likely than athletes with no known prior infection to have an injury from March 2020 to February 2021 (p=0.85). After the start of the March 2020 stay-at-home orders, 31.5% of athletes stated that they were less likely to pursue medical treatment for sport injuries, whereas 14.4% reported being more likely, and 54.1% reported no change. Of the athletes, 50% reported training less from March 2020 to February 2021 compared to the year prior.

## Discussion

This study suggests that the impact of the COVID-19 lockdowns led to a greater incidence of injury in this cohort of collegiate athletes. Half of this cohort also reported training less following the start of the pandemic, which is in accordance with existing research [10]. The etiology behind this injury prevalence increase is unclear and likely multifactorial. However, possible explanations include misjudgment of sport readiness, deconditioning, the lack of access to sport training facilities, and psychosocial factors [11]. A volatile official sports training and competition schedule may have also contributed to this injury increase. Notably, existing literature has associated a higher incidence of injury during the start of a new season following a period of rest (preseason), compared to that occurring during the season [12]. In the year following the start of the COVID-19 pandemic lockdowns, NCAA athletes had to intermittently isolate and withdraw from sports play due to positive COVID-19 testing and fluctuating government and school mandates. This likely artificially created several preseason-like periods throughout the year. Overall, athletes had to juggle additional COVID-19-related stressors including schedule disruptions, concerns about infection, and new COVID-19 protocols or training limitations, which may explain some of the injury results observed in our study.

In addition, in the year prior to and after the start of the COVID-19 pandemic lockdowns, official team practice is where our athletes sustained the greatest number of injuries. This finding fits with the established literature noting official team preseason practice as the setting for high injury incidence. However, additional research examining injury occurrence in relation to time away from sports due to COVID-19 infection or institutional mandates experienced by each athlete is necessary to fully elucidate the role of these shorter sport hiatuses on injury occurrence.

Lastly, a history of laboratory-confirmed COVID-19 infection was not associated with an increased risk of injury. This finding contradicts a previous report that suggested an association between COVID-19 infection and injury [13]. Toresdahl et al. showed a significant increase in the incidence of injury in runners who reported prior COVID-19 infection [13]. However, further investigation is necessary to determine the role, if any, of COVID-19 infection on injury risk.

Limitations

There were several limitations in this study. Fifty percent of all female participants and 26.7% of all respondents were members of the females' track and field and cross-country teams, which may have introduced sampling bias. There is also the possibility of survival bias, whereby those who may have had career-ending circumstances in 2020-2021 would not have been able to participate in this survey. Our use of a survey is also subject to response bias, as participants may not accurately recall their injury history. We also had a relatively small sample size of 146 athletes from one institution, which may not accurately reflect that of the general collegiate athlete population.

## Conclusions

Our study suggests that the COVID-19 lockdowns contributed to the greater injury rates in this group of NCAA Division I athletes. The reasons for this are likely extensive and complex. Athletes also reported training less and having a lower likelihood of pursuing medical intervention for injuries sustained during this time. Lastly, positive COVID-19 infection was not associated with an increased risk of injury. Future research needs to clarify whether the variations in our study results and existing research are due to population diversity, sample sizes, or another cause. In addition, our research points to a need for primary care and sports medicine health professionals to carefully monitor athletes for injuries following time away from sports.
